# Factors Associated with Impairment of Quadriceps Muscle Function in Chinese Patients with Chronic Obstructive Pulmonary Disease

**DOI:** 10.1371/journal.pone.0084167

**Published:** 2014-02-18

**Authors:** Chunrong Ju, Rongchang Chen

**Affiliations:** The First Affiliated Hospital of Guangzhou Medical University, Guangzhou, China; Children's Hospital Los Angeles, United States of America

## Abstract

**Background:**

Quadriceps muscle dysfunction is well confirmed in chronic obstructive pulmonary disease (COPD) and reported to be related to a higher risk of mortality. Factors contributing to quadriceps dysfunction have been postulated, while not one alone could fully explain it and there are few reports on it in China. This study was aimed to investigate the severity of quadriceps dysfunction in patients with COPD, and to compare quadriceps muscle function in COPD and the healthy elderly.

**Methods:**

Quadriceps strength and endurance capabilities were investigated in 71 COPD patients and 60 age-matched controls; predicted values for quadriceps strength and endurance were calculated using regression equations (incorporating age, gender, anthropometric measurements and physical activities), based on the data from controls. Potential parameters related to quadriceps dysfunction in COPD were identified by stepwise regression analysis.

**Results:**

Mean values of quadriceps strength was 46% and endurance was 38% lower, in patients with COPD relative to controls. Gender, physical activities and anthropometric measurements were predictors to quadriceps function in the controls. While in COPD, forced expiratory volume in 1 second percentage of predicted value (FEV_1_% pred), nutritional depletion, gender and physical inactivity were identified as independent factors to quadriceps strength (*R^2^* = 0.72); FEV_1_%pred, thigh muscle mass, serum levels of tumor necrosis factor-alpha (TNF-α) and gender were correlated to quadriceps endurance variance, with each *p*<0.05.

**Conclusion:**

Quadriceps strength and endurance capabilities are both substantially impaired in Chinese COPD patients, with strength most affected. For the controls, physical activity is most important for quadriceps function. While for COPD patients, quadriceps dysfunction is related to multiple factors, with airflow limitation, malnutrition and muscle disuse being the main ones.

## Introduction

Skeletal muscle dysfunction is well documented as an important systemic manifestation of chronic obstructive pulmonary disease (COPD) and has been recognized as a contributing factor in reduced exercise capacity, impaired quality of life and higher health-care utilization. Furthermore, COPD patients exhibit significant reductions in functional mobility and balance that may affect their ability to perform the activities of daily life. It has been suggested that these reductions in functional performance are related to the muscle dysfunction present in these patients [1]. It is of great interest that the pattern of limb muscle impairment in COPD is different from that seen in the respiratory muscles [2]. There is increasing interest in the role of peripheral skeletal muscles in COPD as it is a potential site of intervention for improving the patient's functional status. Among the reports, quadriceps function assessment was used in the majority of the studies for assessment of peripheral muscles' function as it is readily accessible and is a primary locomotor muscle. Quadriceps dysfunction can be considered in terms of loss of both muscle strength and endurance. Current opinion suggests that the reduction in muscle strength is due to a reduction in cross sectional area while the loss of endurance is due to muscle fiber type changes [3]. Quadriceps muscle dysfunction has been reported to be associated with decreased survival [4], poor functional status, and low quality of life [5]. More importantly, quadriceps muscle strength can better predict mortality than measures of lung function in this population [6]. Improved quadriceps muscle strength and endurance are recognized to underlie much of the increased exercise capacity observed following multidisciplinary pulmonary rehabilitation for COPD [7]. Thus, better understanding of the severity and the factors associated with impairment of quadriceps muscle function in patients with COPD would help develop new preventive interventions in quadriceps dysfunction and therapeutic approaches in the rehabilitation of these patients. However, there are few reports on quadriceps dysfunction in China though there are a large population of COPD patients in China [8]. In recent years, factors related to quadriceps dysfunction have been postulated, such as systemic inflammation, muscle wasting, muscle disuse etc.; while no one can fully explain quadriceps muscle dysfunction in COPD. Therefore, this study was aimed at investigating quadriceps dysfunction in Chinese patients with COPD, and to explore the related underlying factors. We examined in detail the quadriceps' function and endurance, the patients' nutritional status, muscle mass and physical activity and the presence of two cytokines; tumor necrosis factor-alpha (TNF-α) and C-reactive protein (CRP) to examine the potential systemic inflammatory response. In addition, the present study aimed to investigate the predictors for quadriceps functional capabilities in the healthy elderly across an age range comparable to that typically observed in COPD.

## Materials and Methods

### Subject selection

The current research was approved by the Research Committee of Human Investigation of Guangzhou Medical University (Approval number: 2011-21). Informed written consent was obtained from each participant. 71 patients with stable COPD were recruited from the outpatients' clinics of Guangzhou Institute of Respiratory Disease (Guangzhou, China) between Mar 2007 to Jun 2009. The diagnosis of COPD was made according to the criteria recommended by the GOLD guidelines [9] with spirometric confirmation of irreversible airflow limitation with post-bronchodilator forced expiratory volume for 1 second (FEV_1_)/forced vital capacity (FVC) <70%. Significant co-morbidities were excluded by medical history, physical check-up and conventional laboratory investigations. All the COPD patients involved in the current study were ex-smokers with abstinence for more than 3 years. Subject exclusion criteria included history of exacerbation in the preceding 3 months, co-morbidities of cardiac, rheumatologic or neuromuscular disorders or unwillingness to participate in the study. Most of the COPD patients were on inhaled corticosteroids (400–800 ug budesonide equivalent dose/day), none of them were on regular systemic corticosteroids otherwise they would have been excluded; about 15% of patients were naïve to inhaled corticosteroids. 60 subjects for the control group were recruited from the health check-up department of First Affiliated Hospital of Guangzhou Medical University. The criteria for inclusion in the control group were as follows: (1) aged matched to the study group with COPD; (2) normal spirometry; (3) without any respiratory symptoms or other disease affecting quadriceps function; (4) non-smoker or has abstained from smoking for more than 10 years.

### Methods

#### Quadriceps function assessment

**Figure 1:**
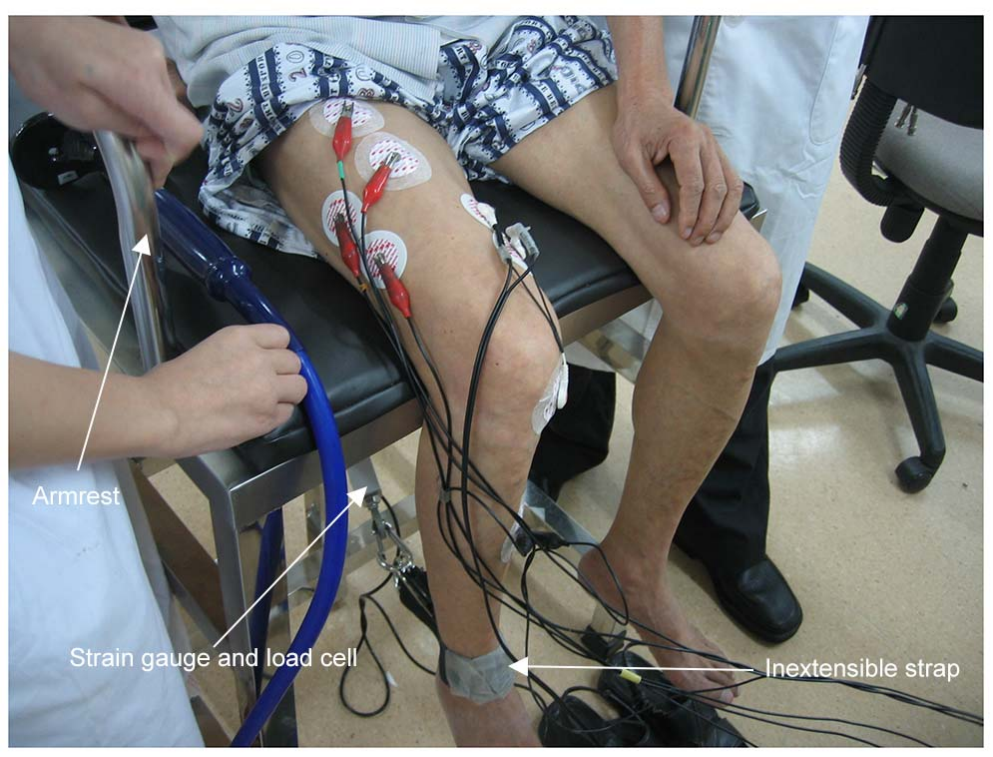
The functional test of quadriceps.

**Figure 2:**
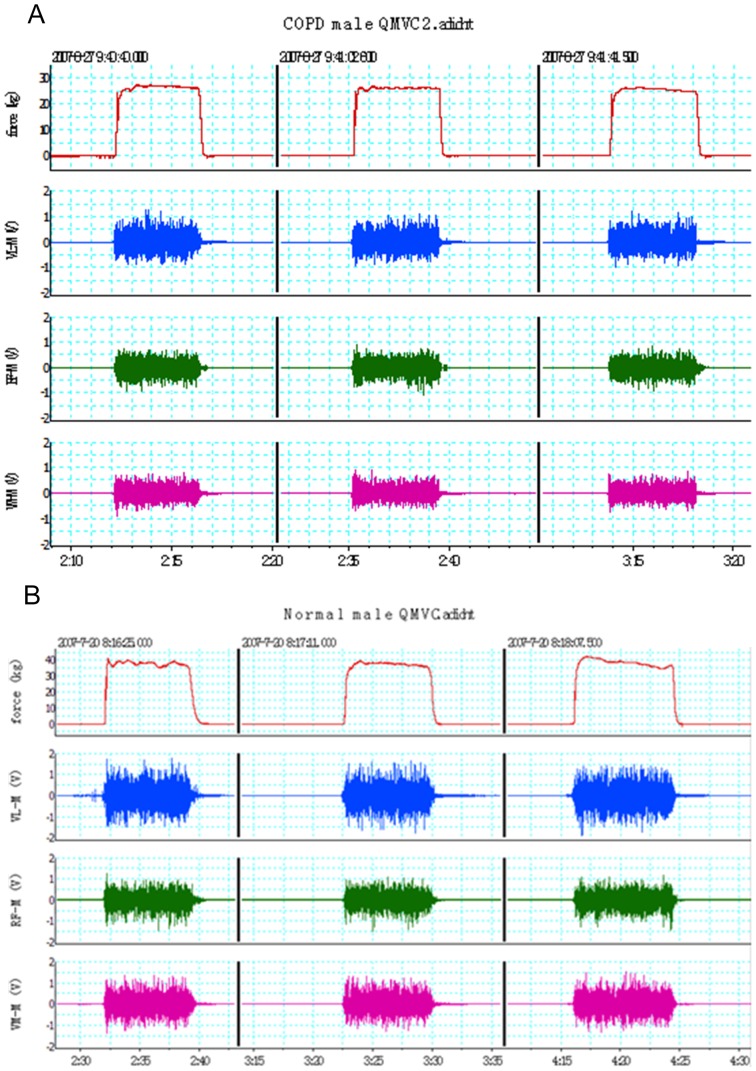
Simultaneous force and surface electromyography (SEMG) recordings during quadriceps maximal volitional contraction (QMVC) tests: the lines on the top channel represent the 3 consecutive volitional contraction forces; SEMG signals from vastus lateralis muscle (VL-M), rectus femoris muscle (RF-M) and vastus medialis muscle (VM-M) were shown in channel 2, 3 and 4, respectively; the number below the X axis represents the time (minute: second). QMVC was about 25(Figure 2A) 40 kg in a male normal subject (Figure 2B).

Quadriceps functional tests included strength and endurance performance. The quadriceps isometric maximal voluntary contraction force (QMVC) test was performed using the technique described by a previous report [10], with a specially designed chair (Figure 1). The chair was designed for the following four functions: first, the chair was immovable with being fixed on the floor, while it was comfortable enough and its armrest was strong enough for subjects to exert their maximal force in a sitting position by wrapping their fingers around the armrest; second, there was a strain gauge and load cell (Strainstall, Cowes, UK) installed under the chair so that the quadriceps force could be measured, and the height of the strain load cell was parallel to the ankle of the subjects; third, the strain load cell could be easily dissembled for everyday calibration; fourth, the back of the chair was movable so it could be changed into a bed by laying the back flat for subjects to lie down if necessary. The tests were performed with the subject in a sitting position at 90° hip flexion and knee flexed at 90° over the end of the chair. An inextensible strap was attached around the subject's right leg just superior to the malleoli of the ankle joint. The strap was connected to the strain load cell that was calibrated after each test with weights of known amounts. Subjects were required to try to extend their dominant leg as hard as possible against the inextensible strap. A computer screen was in front of the subjects in order that the force generated was visible to subjects and investigator, so the computer screen served as a positive feedback to help subjects to perform the test. Repeated efforts were made with vigorous encouragement until there was no improvement in the performance, and each effort was sustained for about 3–5 seconds. If maximal values were reproducible (<10% variability) for a consecutive 3 times, i.e., the generated strength reached a plateau, the highest value of the 3 contractions was considered as QMVC [11]. Surface electromyography (sEMG) was recorded for quadriceps muscles of vastus lateralis (VL), rectus femoris (RF), and vastus medialis (VM). The output signals of force and sEMG were recorded via an analogue-digital instrument (Powerlab 8/16SP Instruments, Austin, TX, USA) and a personal computer (Apple Computer Inc., Cupertino, CA, USA) running Chart 5.1 software. The quadriceps sEMG amplitude recordings were quantified by using the root-mean-square (RMS). Typical signals of QMVC and sEMG from a normal male subject are shown in Figure 2.

#### Endurance time

**Figure 3:**
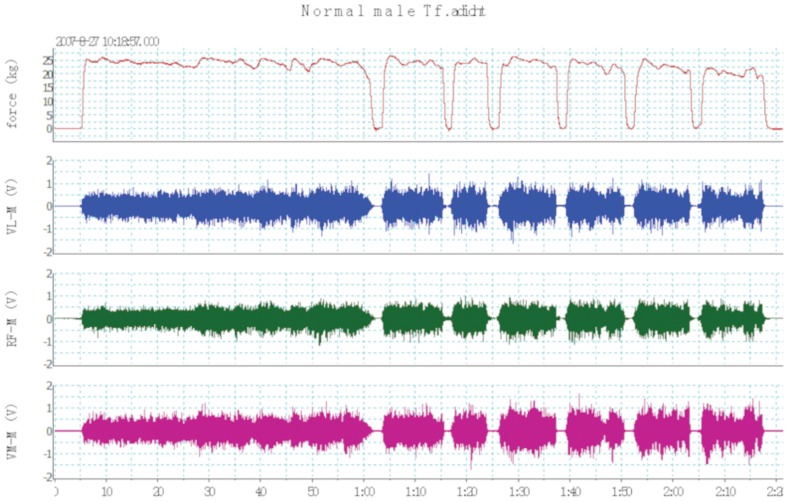
Quadriceps endurance time test: the lines on the top channel represent the sustained isometric contraction at 60% value of quadriceps maximal volitional contraction (QMVC); surface electromyography (SEMG) from vastus lateralis muscle (VL-M), rectus femoris muscle (RF-M) and vastus medialis muscle (VM-M) were shown below the force line; the number below the X axis was the time (minutes: seconds). The time to fatigue (QTf) in this figure was about 2 minutes and 20 seconds (140 seconds).

Endurance of the quadriceps was evaluated during an isometric contraction. After 10 minutes of rest following the QMVC maneuvers, subjects were instructed to maintain a tension representing 55%∼60% of their own QMVC until exhaustion. The feedback mechanism served by the computer screen helped subjects to maintain the determined submaximal tension. Subjects were strongly encouraged to pursue until tension dropped to 50% QMVC or less for more than 3 seconds. Thus, quadriceps endurance was defined as the time to fatigue (QTf), and the time at which the isometric contraction at 60% of maximal voluntary capacity could no longer be sustained. A sample signal from a normal male control is shown in Figure 3.

#### Nutritional status assessment

The nutritional status of all subjects was evaluated by using an integrated approach with a modified multiparameter nutritional index (MNI) [12], which consisted of anthropometric measurements and visceral protein levels. Anthropometric measurements included body weight, triceps skinfold thickness (TSF), mid-arm muscle circumference (MAMC). MAMC = mid-arm circumference –π×TSF. Albumin, transferrin and prealbumin plasma concentration were used as visceral protein levels. The MNI score was calculated by: MNI = a+b+c+d+e+f (0 to15). Table 1 shows the variables and point values used for the computation of MNI score.

**Table 1:** Multiparameter nutritional index for all subjects.

Number	Factor	Range	Score
a	Weight, %IBW	70–89%	3
		<70%	4
b	TSF, %pred	60–79%	2
		<60%	3
c	MAMC, %pred	60–79%	2
		<60%	3
d	Transferrin (g/L)	2.0–1.8	1
		<1.8	2
e	Albumin(g/L)	30–35	1
		<30	2
f	Prealbumin (mg/L)	<200 mg/L	1

IBW: ideal body weight; TSF: triceps skinfold thickness; MAMC: mid-arm muscle circumference; %pred: percentage of predicted.

#### Quadriceps muscle mass evaluation

The quadriceps muscle mass was evaluated indirectly by anthropometric measurements of the legs [13]. Measurements consisted of quadriceps skin-fold thickness (S) and thigh girth of the subjects. With the patient standing and his weight evenly distributed, the thigh girth was determined on the nondominant side at half the distance between the inguinal crease and a point midway along the patella. Thigh muscle mass was thus evaluated as skinfold corrected thigh girth (CTG), and CTG = mid-thigh girth–πS.

#### Determination of cytokines

In serum, levels of TNF-α, and CRP were determined. TNF-α level was determined by ELISA kits (Quantikine; R&D Systems, Minneapolis, MN), and CRP by ELISA kits (CHEMICON Int CO. USA).

#### Level of daily physical activity

The level of daily physical activity (PA) was assessed by using a PA questionnaire [14] adapted for the elderly in China. The questionnaire on habitual PA consisted of 19 items, scored the past 3 year's household activities, sports activities, and other physically active leisure-time activities and gave an overall PA score. An intensity code based on net energetic costs of activities according to Voorrips et al [15] was used to classify each activity. The subjects were asked to describe type of the PA, hours per week spent on it, and period of the year in which the PA was normally performed.

### Statistical analyses

Statistical analysis was performed using SPSS 13.0 (SPSS; Chicago, IL) and Minitab 16.0 (Minitab; Techmax Inc.) statistical package for windows. Measurement data were summarized by mean±*SD*, and categorical data were summarized by number (percentage). *P* value <0.05 was considered statistically significant. Two independent-sample *t*-tests and Chi-square test were used for univariate testing between COPD patients and control subjects. In both control and COPD groups, multiple regression models were developed by stepwise method to determine factors independently contributing to quadriceps strength and endurance, respectively. In the stepwise regression analysis, Alpha-to-Enter 0.15 and Alpha-to-Remove 0.15 were included.

## Results

### Characteristics of the subjects

The general characteristics of the control subjects and patients with COPD are shown in Table 2. Age and height were comparable between the two groups, while differences were observed for body mass index (BMI). Spirometry tests showed that COPD patients had, on average, severe airflow limitation as defined by the GOLD criteria, with FEV_1_ less than 50% predicted. Stratified for the severity distribution, there were 13 (9M and 4F), 28 (22M and 6F), and 30 (23M and 7F) in GOLD stages II, III and IV, respectively.

**Table 2:** General characteristics of the study participants (mean±SD).

Characteristics	Control (n = 60)	COPD(n = 71)	Statistics	*P* values
Sex, M (%)	21 (35%)	54 (76%)	6.34*	0.012
Age, yrs	64.01±5.41	65.17±6.80	0.04	0.30
Height (cm)	161.03±7.80	162.22±7.14	0.91	0.36
BMI(kg/m^2^)	22.641±2.24	19.15±3.19	7.33	<0.001
FEV_1_%pred (%)	97.10±8.90	37.76±14.93	28.10	<0.001
FEV_1_/FVC (%)	88.52±8.41	44.97±12.44	24.31	<0.001
MNI (score)	1.13±0.96	7.76±3.86	13.95	<0.001
PA scores (score)	7.97±1.21	5.05±1.89	12.09	<0.001
Smoking (pack*years)	1.05±4.36	36.70±18.38	8.76	<0.001

*: chi-square value, the others in the colume all represents t values; BMI: body mass index; FEV_1_: forced expiratory volume for 1 second; MNI: multiparameter nutritional index; PA: physical activity.

### Nutritional status and anthropometric data

All nutritional variables were within the reference values in almost all the control subjects, but below the reference values in most of the COPD patients. MNI sore was significantly higher in COPD patients than in controls (table 2). However, CTG were decreased in COPD patients when compared to gender-matched controls [(36.20±3.66) cm *vs.* (29.17±2.49) cm for female, and (42.82±2.49) cm *vs.* (30.46±3.06) cm for male], with each *p*<0.05.

### Level of daily life physical activities

The questionnaire for daily life PA showed that nobody had ever participated in a rehabilitation program among all the participants, while PA scores were significantly lower in COPD patients when compared to controls (table 2), showing a decreased physical activity among the patients.

### Quadriceps functional assessment

As expected, there was a gender-related difference in quadriceps strength; QMVC was significantly decreased in females than in males for both COPD patients and controls. When compared with gender-matched controls, the mean values of QMVC and QTf were both significantly reduced in COPD patients, which were also demonstrated for RMS amplitudes of the sEMG signals from VL, RF and VM muscles during the QMVC test (table 3). The mean value of quadriceps strength and endurance was 46% and 38% lower, respectively, in COPD patients than in controls. Among patients with COPD, there was neither a significant difference in QMVC between the steroid-naive patients and those on inhaled steroids [(24.57±6.41) kg vs. (20.76±6.12) kg, *p* = 0.08], nor was there a significant difference in QTf between them [(51.25±17.38)S vs. (60.56±19.81)S, *p* = 0.15].

**Table 3:** Quadriceps function and RMS of the SEMG during QMVC test (mean±*SD*).

Variables	Sex	Control (n = 60)	COPD (n = 71)	*t* value	*P* values
QMVC (kg)	male	42.06±7.61	23.25±5.70	11.58	<0.001
	female	29.24±4.58	15.55±4.25	10.83	<0.001
QTf (S)	male	81.09±22.58	51.13±18.15	5.87	<0.001
	female	83.44±23.64	56.31±15.49	5.09	<0.001
VL-RMS (mv)	male	405.76±82.52	294.77±89.27	4.93	<0.001
	female	306.13±63.13	218.94±48.95	5.06	<0.001
RF-RMS (mv)	male	334.35±104.72	239.89±89.75	3.90	<0.001
	female	252.19±53.47	173.46±73.72	4.50	<0.001
VM-RMS (mv)	male	345.77±66.56	274.13±81.00	3.60	<0.01
	female	281.52±75.15	188.35±53.51	4.62	<0.001

QMVC: quadriceps maximal volitional contraction; QTf: quadriceps time to fatigue; VL: vastus lateralis; RF-M: rectus femoris; VM: vastus medialis; RMS: root mean squares; RF: rectus femoris; RMS: root mean squares; SEMG: surface electromyography;

### Serum levels of TNF-α and CRP

Serum levels of TNF-α were significantly increased in patients when compared to controls [(6.98±2.50) pg/ml *vs.* (4.49±2.42) pg/ml], with *p*<0.005, while no significant difference was observed in CRP levels between the groups [(3.25±0.37) mg/ml *vs.* (3.42±0.89) mg/ml], with *p* = 0.173.

### Correlations

In normal subjects, multivariate stepwise regression analysis suggested that QMVC was predicted by sex (0.386), PA scores (0.279) and weight (0.305), with R^2^ of 0.61 (*p*<0.0001); for endurance time, PA scores (0.519), CTG (0.374), weight (0.617) and sex (−0.985) were the contributors to QTf variance, with R^2^ of 0.58 (*p*<0.001). Based on the regression analysis results, we derived 2 predictive equations for quadriceps strength and endurance time from the healthy elderly with an age range from 58–76 years old. The equations describing predicted QMVC force (kg) and QTf (S) were, as follows:
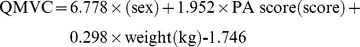


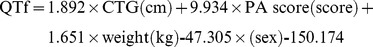



In COPD patients, multiple stepwise regression analysis identified that sex, FEV_1_%pred, MNI and PA scores are statistically significant predictors, together explaining 72% of QMVC variance. For endurance time, FEV_1_%pred, CTG, serum TNF-α levels, and sex were predictors to QTf, explaining 44% of the QTf variance. Table 4 shows the factors correlated with QMVC and QTf in COPD patients. Table 5 and table 6 show the standardized coefficients (*β*) for each predictor variable for QMVC and QTf, respectively, obtained from multiple regression analysis in the COPD group.

**Table 4:** Factors correlated with QMVC and QTf in COPD patients.

		FEV_1_%pred	MNI	PA scores	CTG	TNF-α	sex
QMVC	*R* value	0.325**	−0.279**	0.224**	0.075	−0.08*	0.450**
QTF	*R* value	0.250*	0.200	0.086	0.405**	−0.244**	−0.202*

*: P<0.05;

**: P<0.01; CTG: skinfold corrected thigh girth; FEV1: forced expiratory volume for 1 second; MNI: multiparameter nutritional index; PA: physical activity; QMVC: quadriceps maximal volitional contraction; QTf: quadriceps time to fatigue; TNF-α: tumor necrosis factor alpha.

**Table 5:** Coefficients for each predictor for QMVC obtained from multiple regression analysis.

	Unstandardized	Standardized		P value
	B	Beta	Error	
Constant	−1.928		4.049	0.636
MNI (score)	−0.472	−0.279	0.144	0.002
male/female	6.589	0.450	0.992	<0.001
FEV1pred (%)	0.137	0.325	0.032	<0.001
PA (score)	0.747	0.224	0.270	0.007

FEV1: forced expiratory volume for 1 second; MNI: multiparameter nutritional index; PA: physical activity; QMVC: quadriceps maximal volitional contraction.

**Table 6:** Coefficients for each predictor for QTf obtained from multiple regression analysis.

	Unstandardized	Standardized		P value
	**B**	**Beta**	**Error**	
Constant	12.374		18.85	0.514
CTG (cm)	2.147	0.405	0.610	0.001
TNFa(pg/ml)	−1.757	−0.244	0.660	0.010
FEV1pred(%)	0.302	0.250	0.136	0.030
male/female	−8.479	−0.202	3.843	0.031

CTG: skinfold corrected thigh girth; FEV1: forced expiratory volume for 1 second; QTf: quadriceps time to fatigue; TNF-α: tumor necrosis factor alpha.

## Discussion

The main findings of this study are that (1) Both quadriceps strength and endurance capabilities are substantially impaired in Chinese patients with COPD, with strength and endurance being 46% and 38% lower, respectively, in the patients relative to age-matched controls; (2) Impairment of quadriceps function correlated with multiple factors, with airflow limitation, malnutrition and muscle disuse taking important roles; (3) Using regression equations generated from a cohort of the healthy elderly across an age range from 58 to 76 years old, we showed that physical activity was an important determinant of quadriceps functional capabilities in healthy individuals. As far as the authors know, this is the first study to characterize quadriceps dysfunction in Chinese patients with COPD and give predictive equations for quadriceps strength and endurance time from the Chinese healthy elderly. Also, this is the first study to investigate the multiple factors related to quadriceps function in both COPD patients as well as in the healthy elderly.

In the present study, the expected gender-related difference in quadriceps strength was observed for both COPD patients and controls, and similar data was also reported by Miller et al [16], indicating that gender difference should be taken into account in assessment of quadriceps function. When compared to gender and age-matched controls, the mean value of QMVC was reduced by 47% and 45%, respectively, in female and male patients; the reduction was more severe than previously reported [17], indicating a more remarkable impairment of quadriceps strength in Chinese patients with COPD. But we should elucidate that most of our study patients had severe and very severe airflow limitation. RMS amplitudes of the sEMG signals from VL, RF and VM muscles were also decreased in COPD patients compared to the controls, supporting a significantly decreased strength in the patients, as muscle strength level could be reflected by the amplitudes of the sEMG signals quantified using RMS. For endurance capability, the values of QTf were lowered 39% and 37% in female and male patients, respectively, when compared to controls. Our data showed that quadriceps muscle strength was impaired to a greater extent than endurance in COPD patients, which was in line with the findings of Zattara-Hartmann et al [18]; on the contrary, Van't Hul et al [19] reported greater impairment in endurance for COPD patients. These conflicting results may be attributed to differences in the severity of airflow limitation between the study patients. The patients studied by Van't Hul et al were all in GOLD stage II to III, while most patients in our study were in GOLD stages III to IV. This explanation is supported by the results of regression correlation analysis in the present study, where β coefficient showed that FEV1%pred more significantly correlated with QMVC than with QTf in COPD patients, demonstrating that quadriceps strength is impaired to a larger extent than endurance in COPD. Remarkably, we found that both QMVC and QTf were significantly correlated with FEV1%pred in patients with COPD. Regarding the relationship of airflow limitation with quadriceps function, similar results have been yielded by some previous studies [10], [20], [21], though conflicting results were also reported by other studies [22]–[24]. Nevertheless, the highest prevalence of quadriceps weakness was observed in those with the most severe airflow obstruction in our study patients with COPD, which was also demonstrated in a large sample of COPD patients in another study [17], demonstrating that there may be an association between airflow limitation and quadriceps muscle dysfunction. The relationship between quadriceps dysfunction and airflow limitation may have multiple potential explanations. First, the increased cost of breathing as a result of the airflow limitation may well be associated with skeletal muscle weakness in COPD, especially of the lower limb muscles. Second, airflow limitation and the resultant greater respiratory muscle work often leads to respiratory muscle fatigue, which, in turn, increases sympathetic vasoconstrictor activity in the working limb via a supraspinal reflex [25]. The result is a decrease in limb blood flow and a corresponding reduction in oxygen delivery to peripheral muscles, which accelerate the development of quadriceps fatigue. Third, due to airflow limitation and the associated sensation of dyspnea, COPD patients often experience a downward spiral of symptom-induced inactivity and even muscle disuse, which in turn causes muscle structure changes and metabolic derangements, such as a shift from type I to type II skeletal muscle fibers [26], reduced mitochondrial density per fiber bundle [27], and reduced capillary density [28]. Each of these can correlate with a reduced capacity for aerobic metabolism and, ultimately, poorer muscle performance. In addition, due to airflow limitation and the associated impaired gas exchange, patients with COPD have chronic hypoxia to a varying degree, thus a compromised oxygen transport to limb locomotor muscles might be expected. Furthermore, hypoxemia may interfere with muscle differentiation and lead to muscle dysfunction via several pathways. For example, it has been shown that hypoxia inhibits myogenic differentiation through accelerated MyoD degradation and via the ubiquitin proteasome pathway [29]. In addition, hypoxemia might affect the contractile apparatus and enhance muscle oxidative stress.

As for the nutritional status in the COPD patients, although we recruited the patients randomly at the onset of the study, it turned out that 74.65% patients had decreased body weight, with a mean BMI of less than 21 kg/m^2^ and MNI score that was significantly elevated, indicating that malnutrition was prevalent in the COPD patients; moreover, the MNI score correlated inversely with QMVC in our patients. A similar study result has been already reported [30], and there is evidence that nutritional supplementation increases muscle strength [31]. In our previous study [32], we have found that skeletal muscle mass is substantially decreased in COPD patients and muscle wasting is the main manifestation of nutritional depletion. In the present study, CTG was identified as a contributor to QTf in both COPD patients and controls. This finding was in line with the study result of the association between muscle loss and increased muscle fatigability in COPD [33], suggesting that muscle wasting is, at least in part, responsible for impairment of quadriceps endurance in COPD.

Muscle wasting is an effect of other pathophysiological changes such as muscle disuse and nutritional depletion. At the same time, the present study derived an equation to predict quadriceps strength and endurance, from the healthy elderly; the data showed a close relationship of PA scores with both QMVC and QTf among control subjects. In the classic description of QMVC measurement in COPD, strength was normalized to body weight [34], and in recent research, QMVC was recognized to be associated with airflow limitation, fat-free mass and age [17]. Partly consistent with that study, our data showed that quadriceps strength was predicted by multiple factors including airflow limitation, nutritional depletion, and muscle wasting and physical inactivity. As far as physical activities were concerned, our study found that the PA score was significantly lower in patients than in controls, which was in keeping with previous studies [35], [36]; moreover, our data showed that the PA score had a big effect on both QMVC and QTf in the healthy elderly, based on the standardized β coefficient. Our finding was supported by the classic theory that exercise can improve muscle function while long term inactivity leads to muscle weakness in normal subjects. While in COPD, patients often have a sedentary lifestyle and muscle disuse, which leads to muscle weakness and limited exercise capacity. Our data was also supported by the accumulating study evidence derived from a rehabilitation program, which showed that muscle training can improve muscle strength in COPD patients [37], highlighting a tight link between muscle inactivity and muscle weakness in COPD.

In addition, the present study analyzed the TNF-α levels in serum of the all participants, and found that TNF-α levels were significantly elevated in COPD patients relative to controls; moreover, regression analysis identified TNF-α as one of the contributors to QTf in COPD patients. TNF-α has been recognized as an important cytokine in skeletal muscle wasting [38] as it might compromise muscle function by stimulating muscle protein loss or inducing alterations of muscle proteins catabolism. Our findings, together with previous studies, suggest that systemic inflammation takes an important role in the development of quadriceps dysfunction in COPD. Although one study has found that TNF-α muscle protein levels are decreased in COPD [39] other studies that looked at sputum samples agree with these results [40], [41]. With regard to CRP, our study failed to show a significant difference between COPD patients and the controls. In contrast, Broekhuizen et al [42] reported elevated CRP levels in advanced COPD. CRP is an acute-phase reactant, while patients in our study had been stable for 3 months. This may explain why CRP levels were not elevated in our study.

## Study Limitations

The current study has several limitations. First, we had the small size of female patients with COPD and relatively small size of male controls, which may offset the accuracy of our study results. Larger scale studies should be conducted in the future to further improve the accuracy of the study results. Second, our patients with COPD were on a variety of inhaled corticosteroids (ICS), which might modify the quadriceps function, thereby interfering with the results. Finally, most of the patients in our study had severe or very severe COPD, such that these data cannot be generalized to patients with mild or moderate disease. Further studies are needed to address these issues.

## Conclusions

The present study investigated quadriceps muscle strength and endurance in COPD patients as well as in age-matched healthy elderly; it turned out that the value of quadriceps strength and endurance was 46% and 38% lower, respectively, in COPD patients relative to controls. We draw the conclusion that quadriceps dysfunction is correlated with multiple factors, with airflow limitation, nutritional depletion and muscle disuse taking important roles in its development; while physical activity contributes most to quadriceps function in the healthy elderly.

## References

[1] Butcher SJ, Meske JM, Sheppard MS (2004) Reductions in functional balance, coordination, and mobility measures among patients with stable chronic obstructive pulmonary disease. J Cardiopulmonary Rehabil 24: 274–280. 15286536 10.1097/00008483-200407000-00013

[2] Caron MA, Debigaré R, Dekhuijzen PN, Maltais F (2009) Comparative assessment of the quadriceps and the diaphragm in patients with COPD. J Appl Physiol 107: 952–61. 19359618 10.1152/japplphysiol.00194.2009

[3] Donaldson AV, Maddocks M, Martolini D, Polkey MI, Man WD (2012) Muscle function in COPD: a complex interplay. Int J Chron Obstruct Pulmon Dis 7: 523–535. 22973093 10.2147/COPD.S28247 PMC3430120

[4] Schols AM, Broekhuizen R, Weling-Scheepers CA, Wouters EF (2005) Body composition and mortality in chronic obstructive pulmonary disease. Am J Clin Nutr 82: 53–59. 16002800 10.1093/ajcn.82.1.53

[5] Maltais F, LeBlanc P, Whittom F, Simard C, Marquis K, et al (2000) Oxidative enzyme activities of the vastus lateralis muscle and the functional status in patients with COPD. Thorax 55: 848–853. 10992537 10.1136/thorax.55.10.848 PMC1745616

[6] Swallow EB, Reyes D, Hopkinson NS, Man WD, Porcher R, et al (2007) Quadriceps strength predicts mortality in patients with moderate to severe chronic obstructive pulmonary disease. Thorax 62: 115–120. 17090575 10.1136/thx.2006.062026 PMC2111256

[7] Rabinovich RA, Vilaró J (2010) Structural and functional changes of peripheral muscles in chronic obstructive pulmonary disease patients. Curr Opin Pulm Med 16: 123–133. 20071991 10.1097/MCP.0b013e328336438d PMC2920417

[8] Ju CR, Chen RC (2011) Quadriceps strength assessed by magnetic stimulation of femoral nerve in patients with chronic obstructive pulmonary disease. Chin Med J (Engl) 124: 2309–2315. 21933561

[9] Asia Pacific CRG (2005) Global Initiative for Chronic Obstructive Lung Disease strategy for the diagnosis, management and prevention of chronic obstructive pulmonary disease: an Asia-Pacific perspective. Respirology 10: 9–17. 15691232 10.1111/j.1440-1843.2005.00692.x PMC7169139

[10] Hopkinson NS, Tennant RC, Dayer MJ, Swallow EB, Hansel TT, et al (2007) A prospective study of decline in fat free mass and skeletal muscle strength in chronic obstructive pulmonary disease. Respir Res 8: 25. 17355636 10.1186/1465-9921-8-25 PMC1832189

[11] Vivodtzev I, Pepin JL, Vottero G, Mayer V, Porsin B, et al (2006) Improvement in quadriceps strength and dyspnea in daily tasks after 1 month of electrical stimulation in severely deconditioned and malnourished COPD. Chest 129: 1540–1548. 16778272 10.1378/chest.129.6.1540

[12] Laaban JP, Kouchakji B, Dore MF, Orvoen-Frija E, David P, et al (1993) Nutritional status of patients with chronic obstructive pulmonary disease and acute respiratory failure. Chest 103: 1362–1368. 8486011 10.1378/chest.103.5.1362

[13] Lee RC, Wang Z, Heo M, Ross R, Janssen I, et al (2000) Total-body skeletal muscle mass: development and cross-validation of anthropometric prediction models. Am J Clin Nutr 72: 796–803. 10966902 10.1093/ajcn/72.3.796

[14] Baecke JA, Burema J, Frijters JE (1982) A short questionnaire for the measurement of habitual physical activity in epidemiological studies. Am J Clin Nutr 36: 936–942. 7137077 10.1093/ajcn/36.5.936

[15] Voorrips LE, Ravelli AC, Dongelmans PC, Deurenberg P, Van Staveren WA (1991) A physical activity questionnaire for the elderly. Med Sci Sports Exerc 23: 974–979. 1956274

[16] Miller AE, MacDougall JD, Tarnopolsky MA, Sale DG (1993) Gender differences in strength and muscle fiber characteristics. Eur J Appl Physiol Occup Physiol 66: 254–262. 8477683 10.1007/BF00235103

[17] Seymour JM, Spruit MA, Hopkinson NS, Natanek SA, Man WD, et al (2010) The prevalence of quadriceps weakness in COPD and the relationship with disease severity. Eur Respir J 36: 81–88. 19897554 10.1183/09031936.00104909 PMC3039205

[18] Zattara-Hartmann MC, Badier M, Guillot C, Tomei C, Jammes Y (1995) Maximal force and endurance to fatigue of respiratory and skeletal muscles in chronic hypoxemic patients: the effects of oxygen breathing. Muscle Nerve 18: 495–502. 7739636 10.1002/mus.880180504

[19] Van't Hul A, Harlaar J, Gosselink R, Hollander P, Postmus P, et al (2004) Quadriceps muscle endurance in patients with chronic obstructive pulmonary disease. Muscle Nerve 29: 267–274. 14755493 10.1002/mus.10552

[20] Serres I, Gautier V, Varray A, Prefaut C (1998) Impaired skeletal muscle endurance related to physical inactivity and altered lung function in COPD patients. Chest 113: 900–905. 9554623 10.1378/chest.113.4.900

[21] Coronell C, Orozco-Levi M, Mendez R, Ramirez-Sarmiento A, Galdiz JB, et al (2004) Relevance of assessing quadriceps endurance in patients with COPD. Eur Respir J 24: 129–136. 15293615 10.1183/09031936.04.00079603

[22] Gosker HR, Kubat B, Schaart G, van der Vusse GJ, Wouters EF, et al (2003) Myopathological features in skeletal muscle of patients with chronic obstructive pulmonary disease. Eur Respir J 22: 280–5. 12952261 10.1183/09031936.03.00012803

[23] Degens H, Sanchez Horneros JM, Heijdra YF, Dekhuijzen PN, Hopman MT (2005) Skeletal muscle contractility is preserved in COPD patients with normal fat-free mass. Acta Physiol Scand 184: 235–42. 15954991 10.1111/j.1365-201X.2005.01447.x

[24] Gosker HR, Lencer NH, Franssen FM, van der Vusse GJ, Wouters EF, et al (2003) Striking similarities in systemic factors contributing to decreased exercise capacity in patients with severe chronic heart failure or COPD. Chest 123: 1416–24. 12740256 10.1378/chest.123.5.1416

[25] Dempsey JA, Romer L, Rodman J, Miller J, Smith C (2006) Consequences of exercise-induced respiratory muscle work. Respir Physiol Neurobiol 151: 242–250. 16616716 10.1016/j.resp.2005.12.015

[26] Gosker HR, Zeegers MP, Wouters EF, Schols AM (2007) Muscle fibre type shifting in the vastus lateralis of patients with COPD is associated with disease severity: a systematic review and meta-analysis. Thorax 62: 944–949. 17526675 10.1136/thx.2007.078980 PMC2117111

[27] Gosker HR, Hesselink MK, Duimel H, Ward KA, Schols AM (2007) Reduced mitochondrial density in the vastus lateralis muscle of patients with COPD. Eur Respir J 30: 73–79. 17428811 10.1183/09031936.00146906

[28] Whittom F, Jobin J, Simard PM, Leblanc P, Simard C, et al (1998) Histochemical and morphological characteristics of the vastus lateralis muscle in patients with chronic obstructive pulmonary disease. Med Sci Sports Exerc 30: 1467–1474. 9789845 10.1097/00005768-199810000-00001

[29] Di Carlo A, De Mori R, Martelli F, Pompilio G, Capogrossi MC, et al (2004) Hypoxia inhibits myogenic differentiation through accelerated MyoD degradation. J Biol Chem 279: 16332–8. 14754880 10.1074/jbc.M313931200

[30] Engelen MP, Schols AM, Does JD, Wouters EF (2000) Skeletal muscle weakness is associated with wasting of extremity fat-free mass but not with airflow obstruction in patients with chronic obstructive pulmonary disease. Am J Clin Nutr 71: 733–738. 10702166 10.1093/ajcn/71.3.733

[31] Sugawara K, Takahashi H, Kasai C, Kiyokawa N, Watanabe T, et al (2010) Effects of nutritional supplementation combined with low-intensity exercise in malnourished patients with COPD. Respir Med 104: 1883–1889. 20627502 10.1016/j.rmed.2010.05.008

[32] Ju CR, Chen RC (2012) Serum myostatin levels and skeletal muscle wasting in chronic obstructive pulmonary disease. Respir Med 106: 102–108. 21840694 10.1016/j.rmed.2011.07.016

[33] Kobayashi A, Yoneda T, Yoshikawa M, Ikuno M, Takenaka H, et al (2000) The relation of fat-free mass to maximum exercise performance in patients with chronic obstructive pulmonary disease. Lung 178: 119–127. 10773137 10.1007/s004080000014

[34] Edwards RH, Young A, Hosking GP, Jones DA (1977) Human skeletal muscle function: description of tests and normal values. Clin Sci Mol Med 52: 283–290. 844260 10.1042/cs0520283

[35] Sandland CJ, Singh SJ, Curcio A, Jones PM, Morgan MD (2005) A profile of daily activity in chronic obstructive pulmonary disease. J Cardiopulm Rehabil 25: 181–183. 15931024 10.1097/00008483-200505000-00011

[36] Hernandes NA, Teixeira Dde C, Probst VS, Brunetto AF, Ramos EM, et al (2009) Profile of the level of physical activity in the daily lives of patients with COPD in Brazil. J Bras Pneumol 35: 949–956. 19918626 10.1590/s1806-37132009001000002

[37] O'Shea SD, Taylor NF, Paratz JD (2009) Progressive resistance exercise improves muscle strength and may improve elements of performance of daily activities for people with COPD: a systematic review. Chest 136: 1269–1283. 19734323 10.1378/chest.09-0029

[38] Reid MB, Li YP (2001) Tumor necrosis factor-alpha and muscle wasting: a cellular perspective. Respir Res 2: 269–272. 11686894 10.1186/rr67 PMC59514

[39] Barreiro E, Schols AM, Polkey MI, Galdiz JB, Gosker HR, et al (2008) Cytokine profile in quadriceps muscles of patients with severe COPD. Thorax 63: 100–107. 17875568 10.1136/thx.2007.078030

[40] Warwick G, Thomas PS, Yates DH (2013) Non-invasive biomarkers in exacerbations of obstructive lung disease. Respirology 18: 874–884. 23521049 10.1111/resp.12089

[41] Hacievliyagil SS, Mutlu LC, Temel I (2013) Airway inflammatory markers in chronic obstructive pulmonary disease patients and healthy smokers. Niger J Clin Pract 16: 76–81. 23377476 10.4103/1119-3077.106771

[42] Broekhuizen R, Wouters EF, Creutzberg EC, Schols AM (2006) Raised CRP levels mark metabolic and functional impairment in advanced COPD. Thorax 61: 17–22. 16055618 10.1136/thx.2005.041996 PMC2080712

